# Tight Control of Systolic Blood Pressure in Spontaneous Intraparenchymal Brain Hemorrhage

**DOI:** 10.7759/cureus.5215

**Published:** 2019-07-23

**Authors:** Mark Krel, James Brazdzionis, James G Wiginton, Dan E Miulli, Margaret Rose Wacker, Vladimir Cortez

**Affiliations:** 1 Neurosurgery, Riverside University Health System Medical Center, Moreno Valley, USA; 2 Neurosurgery, Arrowhead Regional Medical Center, Colton, USA; 3 Neurosurgery, Desert Regional Medical Center, Palm Springs, USA

**Keywords:** blood pressure, intraparenchymal hemorrhage, systolic

## Abstract

Background

Tight blood pressure control is critical in neurosurgical patients. Systolic blood pressure (SBP) must be low enough to avoid injury and minimize intraparenchymal hemorrhage (IPH) but high enough to maintain cerebral perfusion. American Heart Association (AHA) guidelines recommend SBP <140 in intracerebral hemorrhage. This paper sought to elucidate the effect of early control of SBP on IPH expansion.

Methods

134 patients with spontaneous IPH between 2011 and 2015 were analyzed utilizing chart review. Initial versus follow-up bleed size, presentation and discharge condition, discharge disposition, and blood pressure control adequacy were analyzed using the generalized linear model.

Results

Altered mental status was the most common presenting complaint (78%). Presenting GCS failed to demonstrate a significant main effect. Age, initial IPH volume, presenting SBP, and one-hour SBP significantly affected IPH percent expansion (p=0.002, =0.002, <0.0005, and =0.026). Several two-way interactions affected IPH percent change implying synergistic effects of the predictor variables.

Conclusion

Patients aged 60-70 years had the largest percent IPH expansion followed by patients aged 20-30 years. Initial IPH volume of 65.23-78.26 ml showed the largest expansion. Initial IPH volume of 52.18-65.22 ml demonstrated the least percentage of IPH expansion. One-hour control of SBP to binned groups of 111-121 mmHg or 121-132 mmHg portends relative minima in bleed expansion corresponding with AHA recommendations for IPH patients. This study suggests that this degree of early and aggressive control of SBP is achievable, safe, and may minimize IPH expansion. Future studies are needed to elucidate the role of co-morbidities and to confirm these findings in broader populations.

## Introduction

Control of blood pressure is a primary concern in neurosurgical procedures and in the medical management of acute stroke. Arterial blood pressure must be kept low enough to avoid injury and minimize bleeding but high enough to maintain cerebral perfusion in the setting of increased intracranial pressure. Since cerebral perfusion pressure (CPP) is equal to the difference between mean arterial pressure (MAP) and intracerebral pressure (ICP), a balance must be maintained between MAP and ICP to ensure adequate CPP. In the case of increased ICP due to events like hemorrhage or mass effect, the elevated ICP leads to compression of cerebral vasculature. This increased cerebral vascular resistance decreases the capillary flow and can lead to cerebral hypoxia and neuronal cell injury, ultimately resulting in death. Normal autoregulation of cerebral blood flow may be disturbed in settings requiring neurosurgical intervention. Therefore, antihypertensive treatment may need to be tailored depending on the specific clinical situation. Patients experiencing stroke have elevated blood pressure in most cases, regardless of stroke mechanism [1, 2]. In acute ischemic stroke (AIS), there is a linear association between SBP and the event of hemorrhage and increased odds of death [2, 3]. In intracerebral hemorrhagic stroke (ICH), the relationship of increased SBP with death and disability also holds true with an increased in-hospital rate of fatality compared to ischemic stroke, and while the rate of disability may be less than in AIS, it is still of grave concern [2, 4, 5].

Hypertension may be directly involved in the secondary expansion of initial hematoma formation [6]. Hematoma expansion (HE) can result in direct mass effect, cerebral edema, and intraventricular hemorrhage leading to hydrocephalus, culminating in increased ICP and decreased CPP [7]. Urgent lowering of blood pressure can minimize hematoma expansion leading to a better outcome [8, 9]. HE is a suitable predictor of poor outcomes based on the modified Rankin Scale (mRS), with at least a fivefold increased risk of clinical neurological deterioration compared to stable hematoma [10-12]. Early HE has been seen in 26% of ICH cases at one hour after admission with a further 12% of patients developing HE by 24 hours [13]. Importantly, neurological deterioration has been shown in 31% of patients with HE vs 10% of patients without, and mRS scores and NIHSS are similarly increased when HE occurs. Prompt reduction of SBP is paramount in maximizing patient outcomes.

Therapeutic SBP and MAP goals have been investigated with mixed results that have yet to demonstrate an absolute target SBP value. Based upon the currently available evidence, the American Heart Association (AHA) recommends reducing SBP to <140 mmHg immediately with SBP <130 mmHg being a reasonable long-term goal to minimize hematoma expansion, increase in ICP, and risk of re-bleeding [14, 15]. These guidelines state that in IPH, CPP should be maintained between 50 and 70 mmHg. Another study suggests CPP <80 mmHg may result in less survivability due to hypoxemia as measured on a brain tissue oxygen sensor, not due to hematoma expansion or re-bleed [16].

Small studies have shown favorable outcomes with lower SBP targets. In a study of 211 patients in whom SBP was reduced to <160 mmHg within one hour of presentation, HE >33% of volume occurred in 17.1% of patients with neurological deterioration in 8.1% of patients [17]. Neurological deterioration was described as a ≥2 decrease in GCS or ≥4 increase in NIHSS score at 72 hr. In this study, 41.2% of patients had an unfavorable mRS score of 4-6 at three-months with 1.9% of patients experiencing mortality by that time. Of note, the average change in the NIHSS score at 72 hours was -2. Overall, reduction of SBP to <160 mmHg showed favorable outcomes and was well tolerated.

In a comparison between nicardipine and nimodipine for hypertensive control between 120 and 140 mmHg within one hour in 87 patients, 21.9% and 18.2% of patients experienced HE >33% at 24 hours, respectively [18]. Neurological deterioration was seen in 28.3% of the nicardipine group and 26.8% of the nimodipine group. A separate study of 88 patients with small-volume ICH and high GCS scores targeted SBP between 100 and 140 mmHg and found only three patients experienced HE, and neurological deterioration was seen in only two patients [19].

 While small studies have shown intensive SBP goals to be tolerable and safe, larger trials have described a somewhat limited effectiveness of intensive goals in long-term outcomes. The Intensive Blood Pressure Reduction in Acute Cerebral Hemorrhage Trial (INTERACT) and the follow-up study, INTERACT2, examined the effect of reducing SBP to <140 mmHg compared with the previously less intensive AHA guideline goal of <180 mmHg [20, 21]. INTERACT found >33% HE in 15% of the intensive group and 23% of the guideline group, demonstrating a 36% lower relative risk of HE in the intensive group. Early neurological deterioration was not different between groups (15% in both) and the 90-day outcomes of mRS, NIHSS, and serious adverse events were also not different. The follow-up INTERACT2 with 2,794 patients showed no difference in mortality or severe disability at 90 days though ordinal analysis did find modestly improved function in the intensive group. Analysis of the INTERACT2 data has shown that SBP reduction >20 mmHg and reduction in SBP in the first hour are associated with lower risk of poor outcomes [9]. This data is additionally summarized below in Table 4 in the discussion.

The Antihypertensive Treatment of Acute Cerebral Hemorrhage (ATACH) study assigned patients into treatment groups with goal SBP of 110-139 mmHg, 140-169 mmHg, and 170-200 mmHg to analyze HE based on the degree of SBP reduction [22]. Greater reductions of SBP were associated with less HE and lower mRS scores at three months. The ATACH2 study of 973 patients targeted intensive goal SBP of 110-139 mmHg and less intensive 140-179 mmHg [23]. Death or disability was found in 38.7% of the intensive group and 37.7% of the less intensive group, demonstrating no significant difference at three months. Together, the INTERACT and ATACH series of studies have suggested that intensive reduction of SBP to <140 mmHg does not pose an increased risk to the patient, and while not reducing mortality, it may provide a small increase in functional outcomes.

Early reduction of blood pressure leads to better outcomes in ICH patients, and the target SBP has not adequately been elucidated up to this point. This study examined 134 patients from 2011 through 2015 with ICH and analyzed the reduction in SBP, time to reduction of SBP, HE, and functional outcomes.

## Materials and methods

One hundred and thirty-four patients seen between 2011 and 2015 at a major county-based hospital for hypertensive intraparenchymal brain hemorrhage were included in this study. Patients who presented for brain hemorrhage but who had underlying pathologic conditions precipitating the hemorrhage such as a mass lesion, vascular lesions, or trauma were excluded from the study. A chart review was performed for each patient who met the inclusion criteria in this study, and the patient's age, ethnicity, presenting complaint, initial IPH size, and blood pressure at one hour were recorded. Size was calculated using the ABC/2 formula for IPH. Then, the size of the IPH on subsequent imaging was recorded. Subsequent imaging was obtained using the standard practice of the neurosurgical center, where-in patients undergo repeat imaging six hours after the initial non-contrast head CT. Lastly, data was collected on the patient's presenting Glasgow Coma Scale Score (GCS) and the patient's GCS at the time of disposition as well as the status of disposition (home, rehab, SNF, other). The data was then analyzed using a generalized linear model with patient age; presenting GCS; initial IPH volume; initial SBP; SBP at one hour; and whether SBP was controlled to < 140, between 140 and 160, or not controlled within one hour. The outcome variable was bleed expansion expressed as a percentage increase or decrease. Wald Chi-Square statistics are reported below. Analysis was completed using SPSS 23.0 (IBM, Armonk, NY). This study was reviewed and approved for chart review by the institutional review board at Arrowhead Regional Medical Center. Due to the nature of the data collection which utilized a chart review, the institutional review board waived the requirement for formal informed consent.

## Results

In total, 134 patients met the inclusion criteria for this study. The mean age of patients was 59.61 years, with a minimum age of 20 years and a maximum age of 91 years. There were 95 males and 92 females. The most common presenting complaint was altered mental status (78%) with severe headache as the second-most common complaint (18%). ICU length of stay ranged from 1 to 34 days with a mean of 6.37 days. Hospital length of stay ranged from 2 to 60 days with a mean of 11 days. Initial presenting GCS ranged from 3 to 15 with a mean of 13.08, while GCS at Day 3 ranged from 8 to 15 with a mean of 13.35. Initial IPH volume ranged from 0.01 ml to 130.43 ml with a mean of 19.6 ml. Follow-up volume ranged from 0.04 ml to 187.44 ml with a mean of 28.63 ml. Percent change ranged from -79% to +1,935% with average change 103.49%. Initial SBP ranged from 104 mmHg to 260 mmHg with mean 166.07 mmHg, and SBP at one hour ranged from 102 mmHg to 209 mmHg with mean 145.79 mmHg. See Table 1 for a summary of this data.

**Table 1:** Summary descriptive statistics of variables. Summary of Abbreviations in Table 1. Length of stay: LOS Intensive care unit: ICU Glasgow Coma Scale: GCS Intraparenchymal hemorrhage: IPH Systolic blood pressure: SBP Standard deviation: Std Dev

Variable	Minimum	Maximum	Mean	Std Dev
Age	20	91	59.61	17.22
LOS ICU	1	34	6.37	7.06
LOS hospital	2	60	11	11.82
GCS initial	3	15	13.08	3.28
GCS Day 3	8	15	13.35	2.46
Initial IPH volume	0.01	130.43	19.6	26.15
Repeat scan IPH volume	0.04	187.44	28.63	39.15
% Change	-79	1935	103.49	268.94
SBP initial	104	260	166.07	32.03
SBP 1 hr	102	209	145.79	23.62

**Figure 1:**
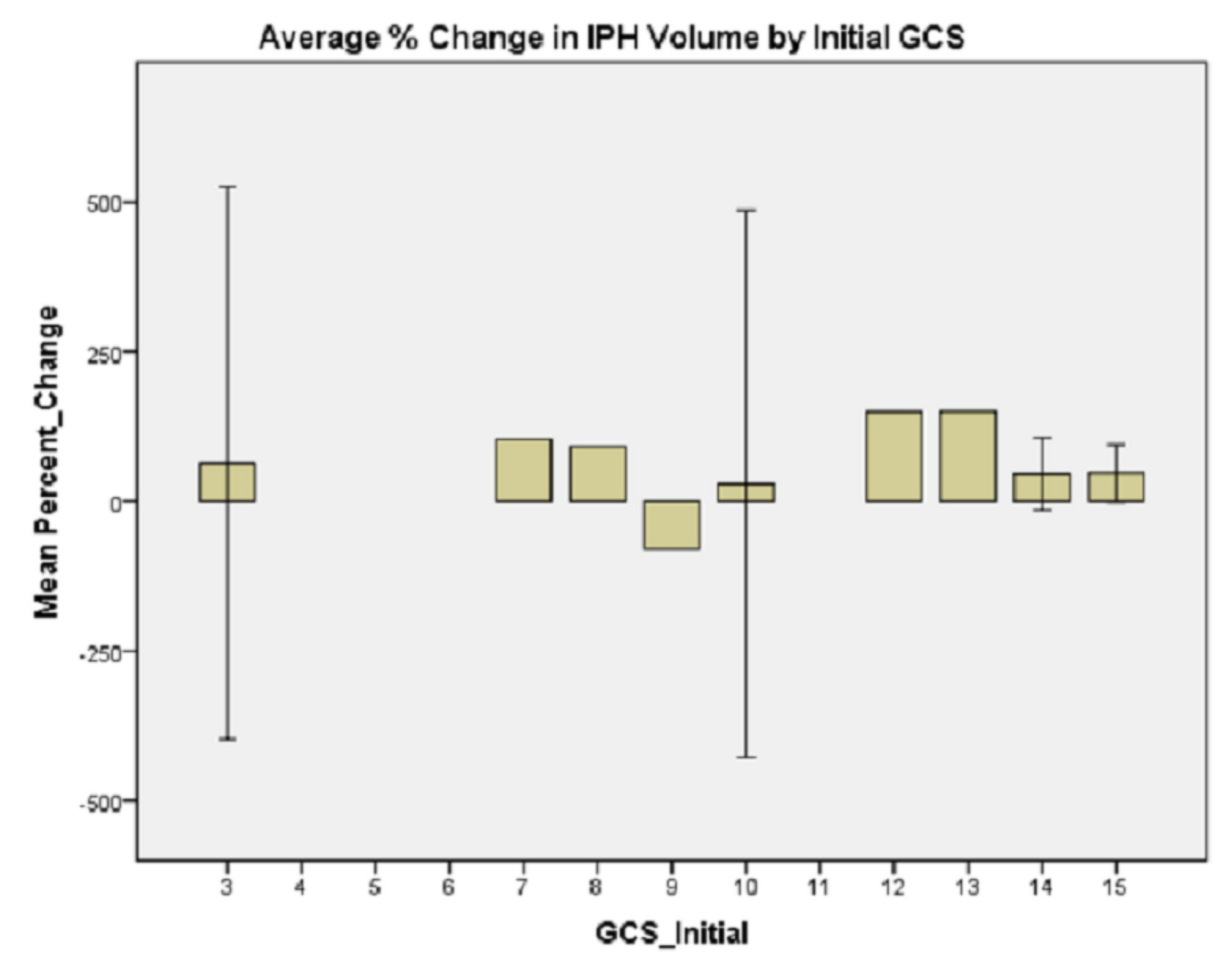
Average % change in IPH volume by initial GCS. While patients who presented with GCS 10 had, in absolute value, that smallest change in IPH volume, the variance was again quite high and patients whose initial GCS was nine seemed to have an overall decrease in IPH size on follow-up imaging. Intraparenchymal hemorrhage: IPH Glasgow Coma Scale: GCS

**Figure 2:**
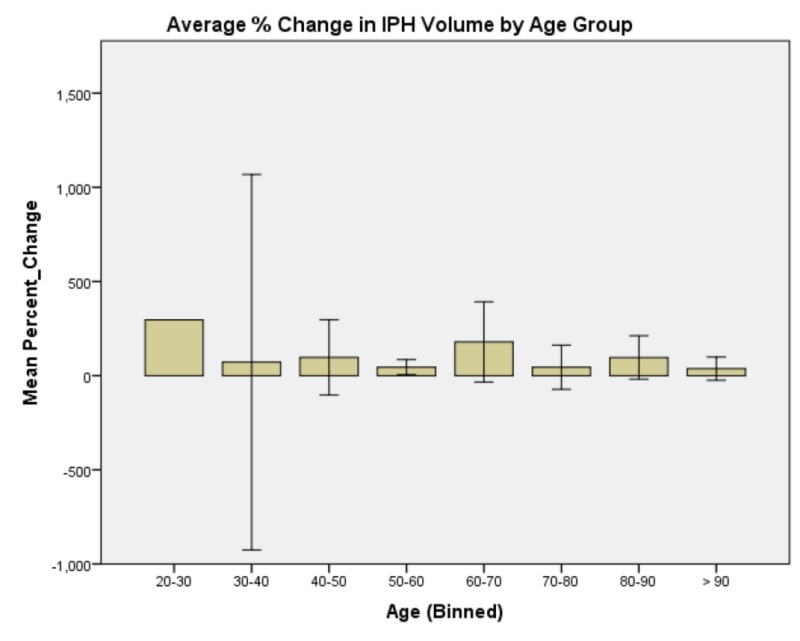
Average % change in IPH volume by age group. Patients in the 60-70 years age group tended to have the largest IPH expansion followed by the 20-30 years age group. Intraparenchymal hemorrhage: IPH

**Figure 3:**
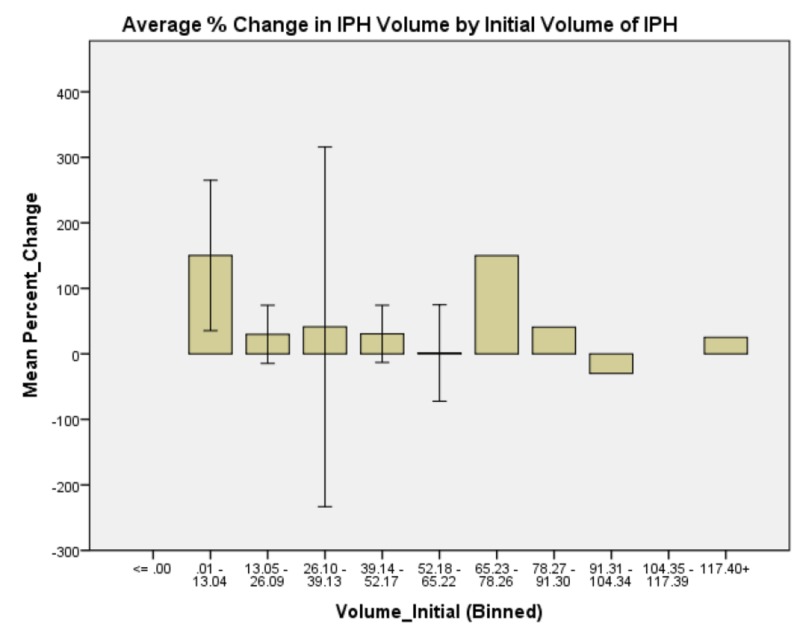
Average % change in IPH volume by initial volume of IPH. Again, the data is roughly normally distributed with peak expansion occurring in the group of patients who presented with initial IPH volume of 65.23 to 78.26 ml. There is also a second maximum in patients who presented with initial IPH volume of 0.01 to 13.04 ml. Intraparenchymal hemorrhage: IPH

**Figure 4:**
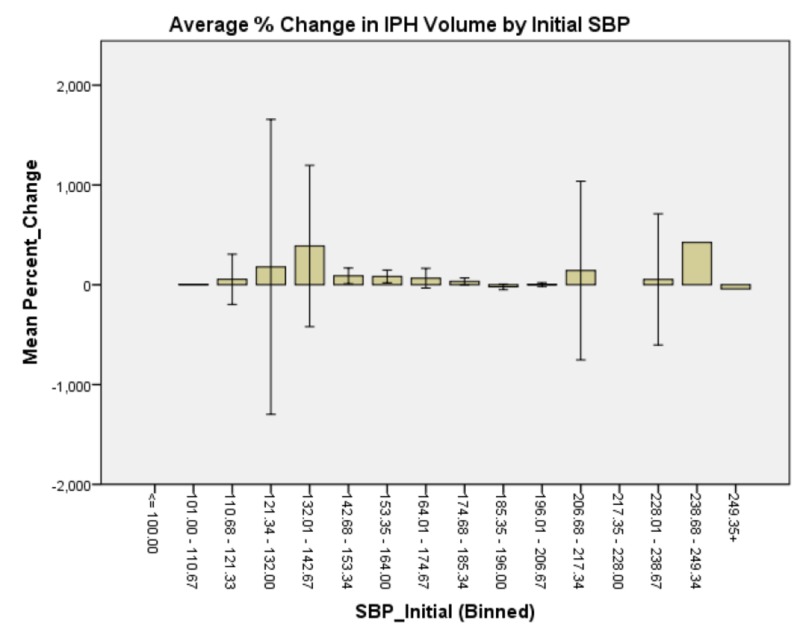
Average % change in IPH volume by initial SBP. There is a bimodal distribution of % change in IPH size by initial SBP, with the largest expansion occurring in patients whose presenting SBP was 132-143 and those at 239-250. Intraparenchymal hemorrhage: IPH Systolic blood pressure: SBP

**Figure 5:**
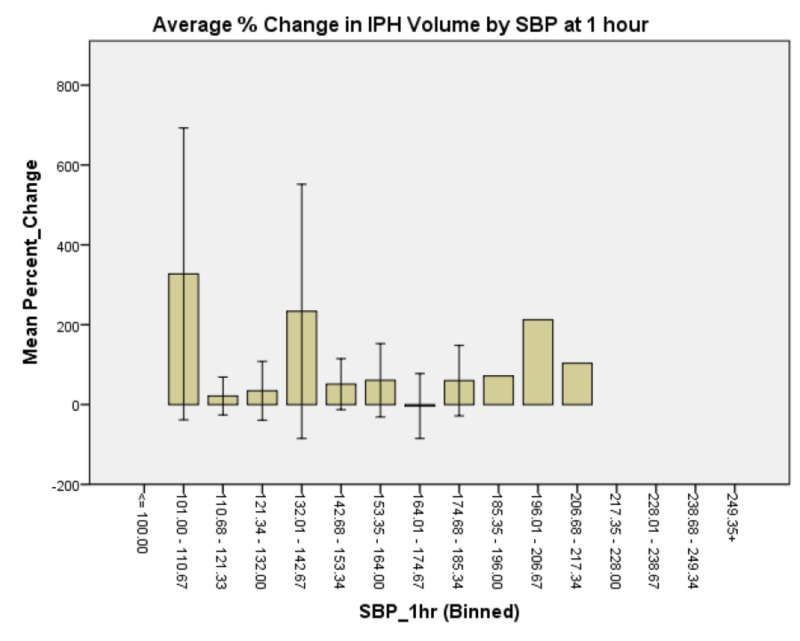
Average % change in IPH volume by SBP at one hour of presentation. Patients whose SBP fell between 132 and 143 again had larger IPH expansions. Of note, the largest IPH expansion occurred with SBP 101-111 and there was another relative maximum at 196-207. Summary of Abbreviations in Figure 5: Intraparenchymal Hemorrhage: IPH Systolic Blood Pressure: SBP

**Figure 6:**
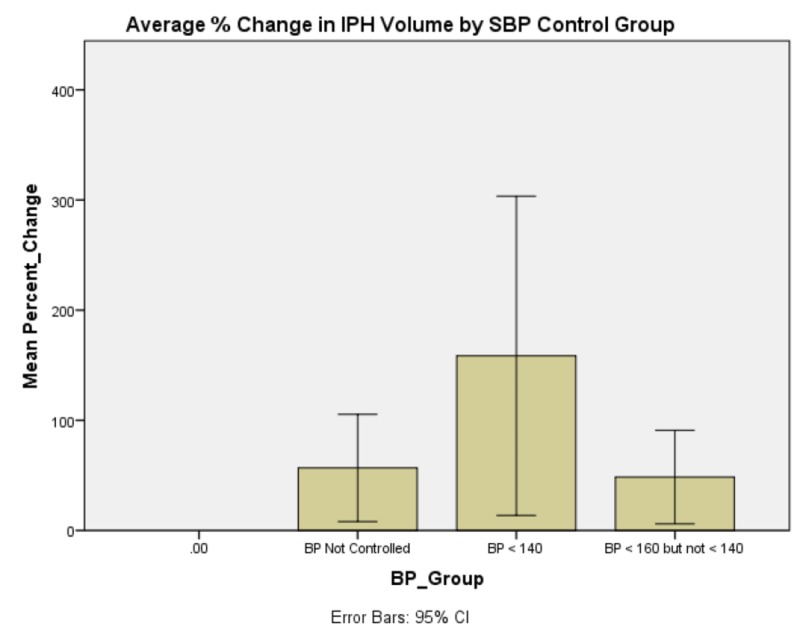
Average % change in IPH volume by SBP control group. Patients whose SBP was not controlled to < 160 and patients whose SBP was controlled to < 160 but not < 140 appeared to have smaller IPH expansion than patients whose SBP was controlled to < 140. There is, however, a large degree of variance in the data analyzed in this way. Summary of Abbreviations in Figure 6: Intraparenchymal Hemorrhage: IPH Systolic Blood Pressure: SBP Blood Pressure: BP

Generalized linear model analysis was performed on the data set with patient age; presenting GCS; initial IPH volume; initial SBP; SBP at one hour; and whether SBP was controlled to less than 140, between 140 and 160, or not controlled to less than 160 within one hour. The outcome variable was hematoma expansion expressed as a percentage increase or decrease. Initial presenting GCS demonstrated no significant main effect on percent change in IPH size, χ2 (6) = 4.958, p = 0.550 (Figure 1). Patient age demonstrated a significant main effect on percent change in IPH size, χ2 (5) = 18.830, p = 0.002 (Figure 2). Initial IPH volume demonstrated a significant main effect on percent change in IPH size, χ2 (3) = 14.472, p = 0.002 (Figure 3). Presenting SBP demonstrated a significant main effect on percent change in IPH size, χ 2 (10) = 34.230, p < 0.0005 (Figure 4). SBP at one hour of presentation demonstrated a significant main effect on IPH size, χ 2 (7) = 15.931, p = 0.026 (Figure 5, Table 2). Notably, if SBP control was binned by level (i.e., analyzed by three groups where Group 1 was patients whose SBP was controlled to < 140 mmHg, Group 2 was patients whose SBP was controlled to < 160 mmHg but not < 140 mmHg, and Group 3 was comprised of the remaining patients whose SBPs were not successfully controlled), no significant main effect was discovered, χ2 (2) = 5.063, p = 0.080 (Figure 6). Table 3 summarizes the factors that affected IPH expansion.

**Table 2:** Summary of mean percent IPH expansion by SBP range at one hour of presentation. Systolic blood pressure: SBP Intraparenchymal hemorrhage: IPH

SBP (mmHg) at 1 hr	Mean % IPH expansion
101-110	411.5
111-121	34
122-132	45.1
133-142	268
143-153	51.1
154-164	60.9
165-175	-4
> 175	59.7

**Table 3:** Summary of factors that significantly affect IPH expansion, the two ranges of maximal expansion, and the percent expansion relatable to that range. Summary of Abbreviations in Table 3: Intraparenchymal Hemorrhage: IPH Systolic Blood Pressure: SBP

Factors Significantly Predicting Maximal IPH Expansion		% Expansion of IPH
Patient Age	20 - 30 years	238.4
	60 - 70 years	196.9
Initial IPH Volume	65.23 - 78.26 ml	168.9
	0.01 - 13.04 ml	157.3
Presenting SBP	133-142 mmHg	584.3
	> 238 mmHg	761.8
SBP at 1 hr	101-110 mmHg	411.5
	133-142 mmHg	268

Factorial analysis revealed several significant two-way interactions. The interaction of age by BP group (as described above) was significant, χ2 (2) = 231.962, p < 0.0005. The interaction of initial SBP with initial IPH volume was significant, χ2 (3) = 171.443, p < 0.0005. The interaction of SBP at one hour of presentation and initial IPH volume was significant, χ2 (2) = 288.190, p < 0.0005. Finally, the interaction of initial SBP and SBP at one hour of presentation was significant, χ2 (1) = 121.677, p < 0.0005.

## Discussion

The data presented in this study exhibit several important findings. Patients in the 60-70 years age bracket tended to have the largest percent IPH expansion followed by the 20-30 years age group. This may be accounted for by the notion that patients age 20-30 years who present with IPH are likely to have inherent predisposition either epigenetically or by exogenous factors such as drug abuse to stroke. Moreover, patients in the 60-70 years group are known to be at high risk for stroke. Patients older than this group may, in fact, be likely to overcome intraparenchymal hemorrhage by inherent predispositions to vascular and parenchymal stability - and this may have conferred the survival advantage that has allowed them to reach advanced ages.

Initial volume of IPH on presentation also predicts IPH expansion with a group of patients who presented with initial IPH volume of 65.23 to 78.26 ml. There is also a second maximum in patients who presented with initial IPH volume of 0.01 to 13.04 ml. The expansion of the IPH in the group presenting initially with the smallest IPH is perhaps unsurprising as any expansion, even layering of blood, would be, in relative quantity, a large percentage change. Of more interest and clinical import, is the finding that patients who present with an initial bleed size between 65.23 and 78.26 ml have predictably larger IPH expansion on follow-up imaging. These patients, therefore, may require closer monitoring and more stringent control of secondary factors such as coagulopathy, blood pressure, cholesterol, and cerebral perfusion pressure. Notably, patients whose initial IPH size was between 52.18 ml and 65.22 ml demonstrated a nadir in IPH expansion. This may perhaps represent the boundary of the brain parenchymal ability to compensate for hemorrhagic insult.

Taken in isolation, it would be easy to conclude that there exists a trend that patients whose SBP was controlled to < 140 mmHg after IPH had larger IPH expansion than patients whose SBP was controlled to between 140 and 160 mmHg (whose expansion was comparable to patients in whom SBP was not successfully controlled). A more nuanced approach, however, demonstrates that while patients did, in fact, have larger bleed expansion in the 132-143 mmHg managed group, patients whose SBP control fell lower than this bin, namely the 111-121 mmHg and 122-132 mmHg groups had relative minima in IPH expansion. This coincides with the AHA recommendation of ultimate SBP control in the face of IPH; however, this data suggest that if this control can reasonably be achieved early in the course of the disease, it may lead to smaller IPH expansion and, by extension, improved clinical outcomes. The relative maximum of bleed expansion in the 101-111 mmHg group may be attributable to derangements in CBF autoregulation that correspond to the low-normal SBP state experienced by these patients.

Future work in this arena may include patients with IPH from our other major centers to expand the sample size and to better approximate the population. Furthermore, future investigation may focus on patient-specific factors in future analyses such as comorbid conditions and confounding factors such as drug use and any potential coincident illnesses such as ventilator-associated pneumonia, cancers, etc. It is well studied that control of systolic blood pressure is critical in patients with intraparenchymal hemorrhage and tight control of blood pressure is a primary concern in the neurosurgical patient. A balance must be achieved wherein the systolic blood pressure is kept low enough to avoid injury and minimize bleeding while simultaneously being kept high enough to maintain perfusion, particularly in the setting of elevated intracranial pressure. Normal autoregulation of cerebral blood flow may be disturbed in pathologic states such as trauma, tumor, or hemorrhagic stroke, and therefore, exogenous control is often required in the neurosurgical patient. Current American Heart Association (AHA) guidelines recommend the maintenance of systolic blood pressure to less than 140 mmHg in the setting of intracerebral hemorrhage with a long-term goal of SBP < 130 mmHg [14]. Table 4 below summarizes several additional studies with blood pressure parameters, IPH expansion, and neurologic decline in their patient cohorts [2, 3, 10, 13, 14, 17, 18].

**Table 4:** Summary of additional studies with regards to SBP targets and outcomes in patients with IPH Intraparenchymal hemorrhage: IPH Systolic blood pressure: SBP Intensive Blood Pressure Reduction in Acute Cerebral Hemorrhage Trial: INTERACT Antihypertensive Treatment of Acute Cerebral Hemorrhage study: ATACH

Study	SBP target	% Hematoma expansion	% Early neurologic deterioration
Koga et al. (N = 211) [17]	<160	17.1	8.1%
Li et al (N = 87) [18]	<140	21.9% (Nicardipine), 18.2% (Nimodipine)	28.3% (Nicardipine), 26.8% (Nimodipine)
Hwang et al (N = 88) [19]	<140	3.4%	2.3%
INTERACT (N = 404) [20]	<140	15%	15%
INTERACT2 (N = 2794) [21]	<180	26.1%	17.9%
ATACH (N = 60) [22]	170-200 vs 140-170 vs < 140	33% vs 15% vs 32%	5.5% vs 10% vs 18%
ATACH2 (N = 973) [23]	<<140 vs <180	18.9% vs 24.4%	11% vs 8%

As mentioned, careful balance is key. Increased intracranial pressure results in elevated interstitial hydrostatic pressure and compression of the cerebral vasculature. This results in decreased capillary flow hydrostatic gradient and increased cerebral vascular resistance. Further, this can lead to cerebral hypoxia and deficient metabolic reserve causing neuronal cell injury and death. Elevated mean arterial pressure (MAP) in the setting of hypertension leads to increased cerebral perfusion pressure (CPP) and can cause endothelial damage with the destruction of the blood-brain barrier and vasogenic edema leading to further intracranial hemorrhage.

## Conclusions

Intraparenchymal hemorrhage is less common than ischemic stroke but has higher morbidity and mortality rates per incident. Therefore, urgent reduction in blood pressure can minimize hematoma expansion leading to a better outcome. Hematoma expansion can result in direct mass effect, cerebral edema, and intraventricular hemorrhage that leads to hydrocephalus. This leads to increased intracranial pressure and decreased cerebral perfusion pressure. At this time, nicardipine is the consensus intravenous continuous infusion drug of choice for emergent control of refractory hypertension; however, nimodipine, labetalol, esmolol, and other drips have been used with varying degrees of efficacy, complications, and clinical outcomes. The patient populations with the largest hematoma expansion are those who were 60-70 years and 20-30 years old and those whose initial ICH volume was 65.23 to 78.26 ml. While our data suggest that the ideal target range for SBP in the setting of acute IPH may be 121 - 132 mmHg, further evaluation of this patient population is required to definitively elucidate a target blood pressure range for the immediate post-insult period in intraparenchymal hemorrhage.
